# Robotic-assisted approach for complex cholecystectomies

**DOI:** 10.20517/2574-1225.2022.117

**Published:** 2023-04-11

**Authors:** Gina Zhu, Kelli Ann Ifuku, Kimberly S. Kirkwood

**Affiliations:** Department of Surgery, University of California, Genentech Hall, San Francisco, CA 94158, USA.

**Keywords:** Robotic-assisted laparoscopic cholecystectomy, indocyanine green, complex cholecystectomy

## Abstract

Robotic approaches have facilitated the minimally invasive completion of increasingly complex surgical procedures. In the management of the difficult gallbladder, we have found that the wristed instruments, three-dimensional camera, the ability to use indocyanine green (ICG) with integrated fluorescent imaging, and ease of intracorporeal suturing to be useful in tackling the challenges associated with complex benign gallbladder disease. We describe the rationale and technical lessons learned during four cases of complex cholecystectomies that highlight the management principles and technical advantages afforded by the use of the robotic-assisted laparoscopic (RAL) approach. The cases include a subtotal fundus-first reconstituting cholecystectomy, subtotal fenestrating cholecystectomy, a cholecystocolonic fistula managed by a RAL subtotal fenestrating cholecystectomy, and an iatrogenic cholecystoduodenal fistula managed by RAL cholecystectomy. In each case, the operation was performed safely without intraoperative injury or conversion to open, and three of the four patients were discharged from the recovery room. In our view, these favorable outcomes were greatly facilitated by the robotic platform. It is our intent to share adaptations and innovations that we found helpful to encourage other surgeons with sufficient robotic experience to tackle complex gallbladder cases minimally invasively.

## INTRODUCTION

Cholecystectomy represents one of the most common surgical procedures, with more than 500,000 performed minimally invasively annually in the US, according to the Society of American Gastrointestinal and Endoscopic Surgeons (SAGES)^[1]^. Although cholecystectomies are extremely common, the procedure is not always simple. Inflammation and ambiguous or distorted anatomy are the hallmarks of a “Difficult Gallbladder” and these qualities have been correlated with higher postoperative complications including bile duct and vascular injuries that can be life-altering^[2]^.

Published strategies to address these risks focus on obtaining a critical view of safety (CVS) and liberal use of cholangiography^[3-5]^. We and others have published modifications and alternatives to “routine” total cholecystectomy, including a modified fundus-first subtotal approach, posterior infundibular, antegrade approaches, and subtotal fenestrating and reconstituting cholecystectomy^[6-10]^. As every operation is different, surgeons must have the knowledge of different techniques to move through their operation safely. There are several avenues to pursue when faced with a difficult gallbladder. Conversion to open cholecystectomy provides haptic feedback but does not always improve visualization of the anatomy. As a result, the expectation that conversion will turn a difficult gallbladder into a straightforward case is frequently not realized. Simply aborting the procedure or placing a cholecystostomy tube are options that can delay the timing of definitive management and may be appropriate in the setting of severe acute inflammation or to defer the case to a more experienced surgical team^[11]^. Subtotal cholecystectomy, where a portion of the gallbladder is removed and a small remnant is either left open (fenestrating) or closed (reconstituting) with or without internal closure of the cyst duct, is often preferred as it provides durable management^[5,12]^.

The use of robotic instruments has the potential to bring specific value to complex or difficult cases. In our experience, we have found the use of the wristed instruments, three-dimensional camera, and the ability to use indocyanine green (ICG) with integrated fluorescent imaging to be particularly useful in tackling the challenges associated with resecting difficult gallbladders. Here we describe four cases of complex cholecystectomies that highlight the algorithms and principles of approaching the difficult gallbladder using a robotic-assisted laparoscopic (RAL) approach.

## CASE REPORT

### Case presentation 1: RAL fundus-first subtotal reconstituting cholecystectomy

A 62-year-old female patient presented with a week of nausea, vomiting, epigastric pain, anorexia, fever, elevated liver function tests (LFTs), and ultrasound demonstrated choledocholithiasis without evidence of cholecystitis. At the time of Endoscopic Retrograde Cholangiopancreatography (ERCP), she was found to have three common bile duct (CBD) stones and underwent sphincterotomy and balloon sweep with stone removal. She then underwent RAL cholecystectomy.

Per our usual routine, every patient is given 2.5-5 mg of indocyanine green (ICG) IV prior to induction. We employ liberal use of indocyanine green, near-infrared (NIR) fluorescence cholangiography and robot-integrated fluorescent imaging to image the biliary system. Since this procedure requires a specimen extraction site, we prefer a longitudinal intraumbilical incision, which provides the best cosmesis. The abdomen is insufflated with a Veress needle at the umbilicus and entered with an 8 mm bladeless robotic trocar and optical obturator. This port, for the second robotic arm, is changed to a 12 mm port under direct vision after the placement of an 8 mm port at the level of the umbilicus on the right for the first robotic arm. This approach avoids harm caused by the attempted insertion of the blunt 12 mm robotic port. Under direct laparoscopic visualization, a third 8 mm port is placed at the level of the umbilicus in the left midclavicular line, and the last 8 mm port is placed above the left anterior superior iliac spine. After the robot is docked, the camera is placed in arm 2, the 12 mm port, for most of the procedure, then moved to arm 3 for extraction of the specimen.

**Figure 1.**
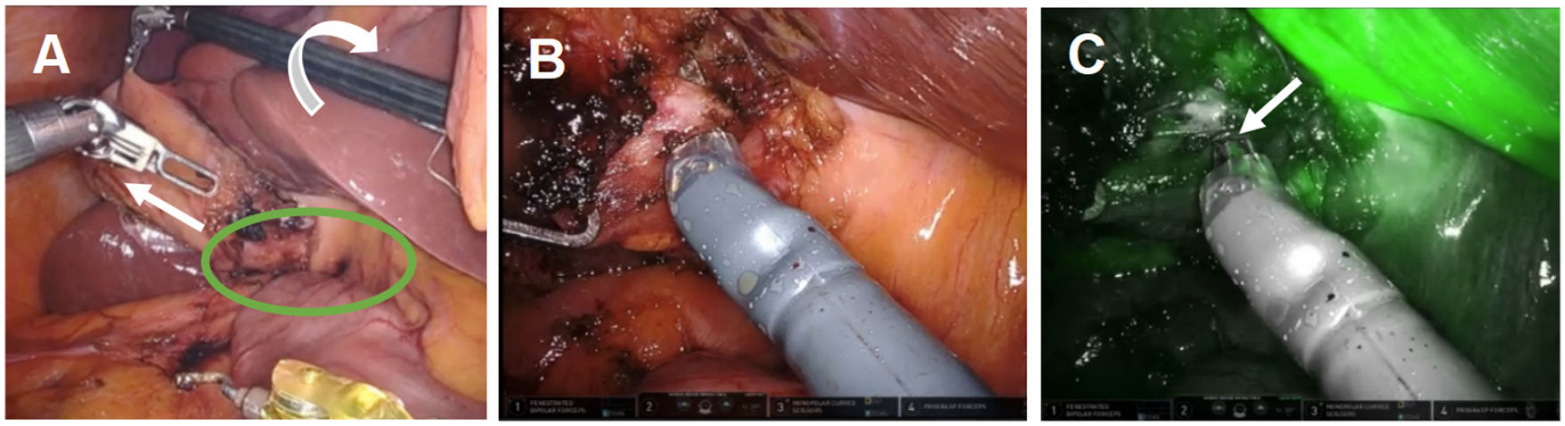
Robotic instruments helped identify biliary anatomy. (A) The wristed instrument enabled clockwise rotation of the fundus (white arrows) to displace the infundibulum anteriorly fused between the infundibulum and common hepatic duct (green oval); (B) use of white light illumination; and (C) near-infrared fluorescence cholangiography with indocyanine green identified the absence of bile in the putative cystic artery (white arrow).

In accordance with the principles of the SAGES Safe Cholecystectomy Program, we first sought to determine whether the CVS could be identified. We firmly grasped the thickened fundus and rolled it clockwise toward the right shoulder to anteriorly displace the infundibulum [Figure 1A]. We identified the common hepatic duct (CHD), confirmed the absence of bile flow in the putative cystic artery, and confirmed one, and only one, bile containing tube exiting the gallbladder. The infundibulum was difficult to manipulate and seemed tethered to the CHD. Based on the possibility that the cystic artery was contributing to our inability to manipulate the infundibulum, we elected to take it first. Identification of the cystic artery was confirmed by its proximity to Calot’s node, the observation that it bifurcated onto the gallbladder wall, its caliber, and the absence of ICG. [Figure 1B and C] The cystic artery was clipped with a large plastic clip and divided.

**Figure 2.**
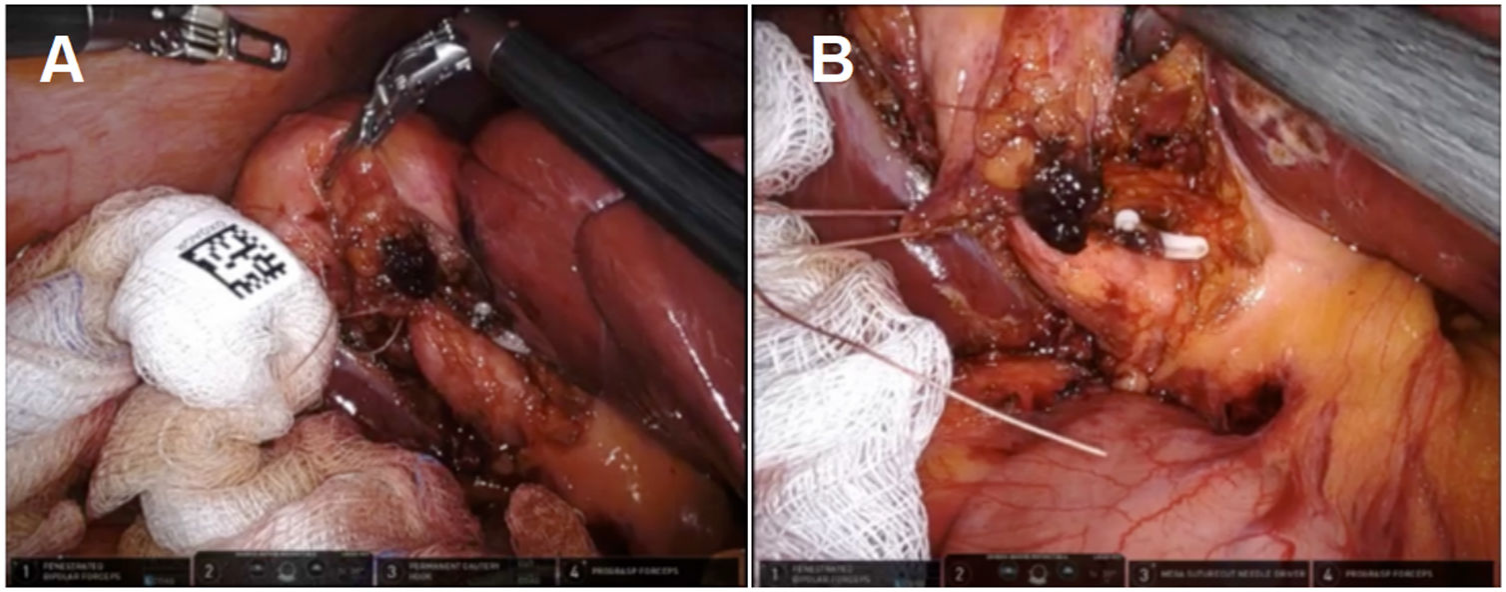
A distended infundibulum with poor mobility was visualized by (A) fundal retraction using the wristed instruments and (B) lateral retraction with 0-vicryl.

We tied a 0-vicryl tie around the infundibulum to facilitate sufficient lateral retraction and confirmed that the posterior extent of the infundibulum, distended by stones, was drawn medially, and appeared to be fused to the right side of the CHD. Using ICG fluorescent cholangiography dynamically, it appeared that the infundibulum was distended by many large stones and moved with the CHD as one [Figure 2].

**Figure 3.**
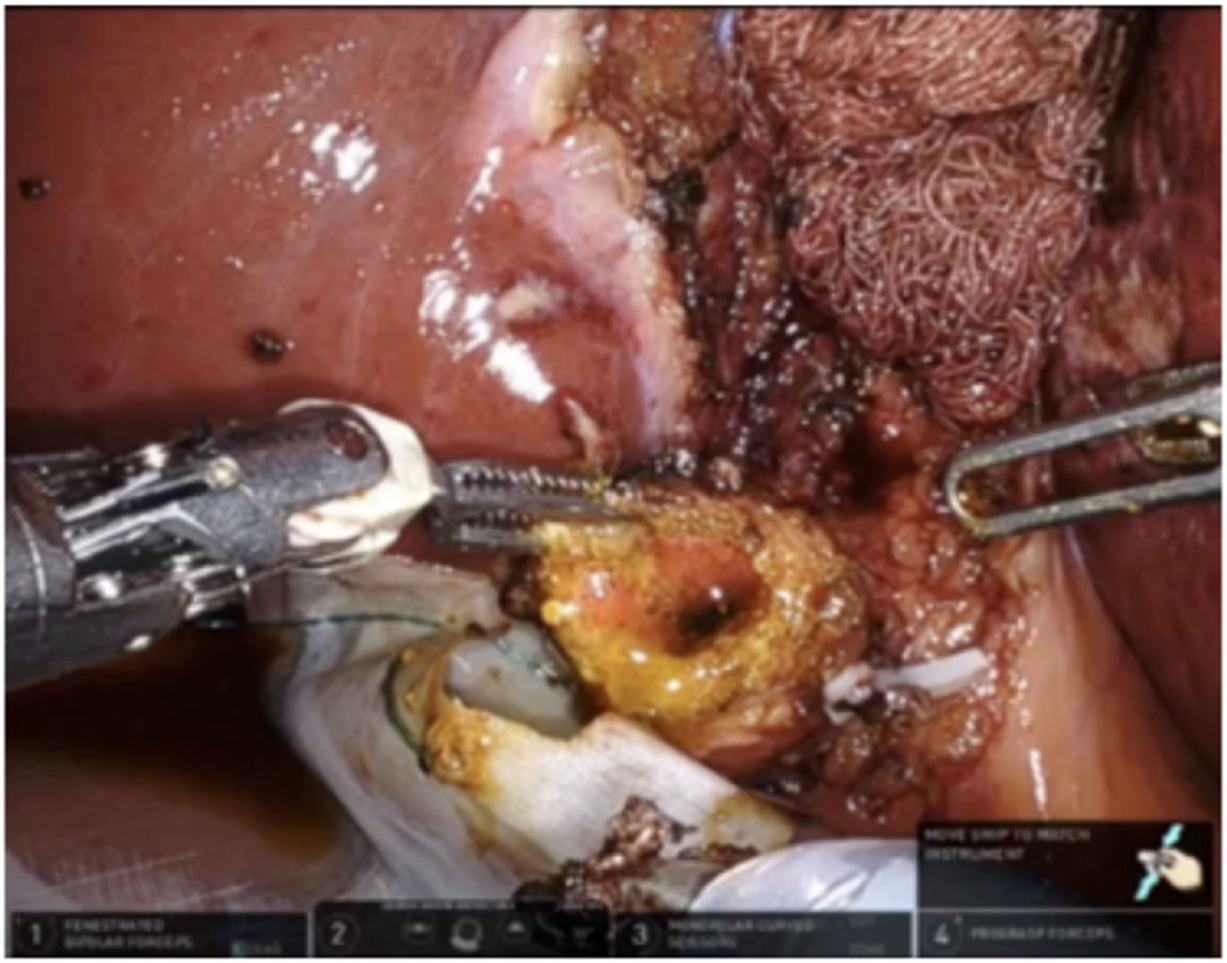
Clear bile was identified emanating from the cystic duct orifice, suggesting the creation of a biliary fistula if left open. A reconstituting approach was thus taken.

**Figure 4.**
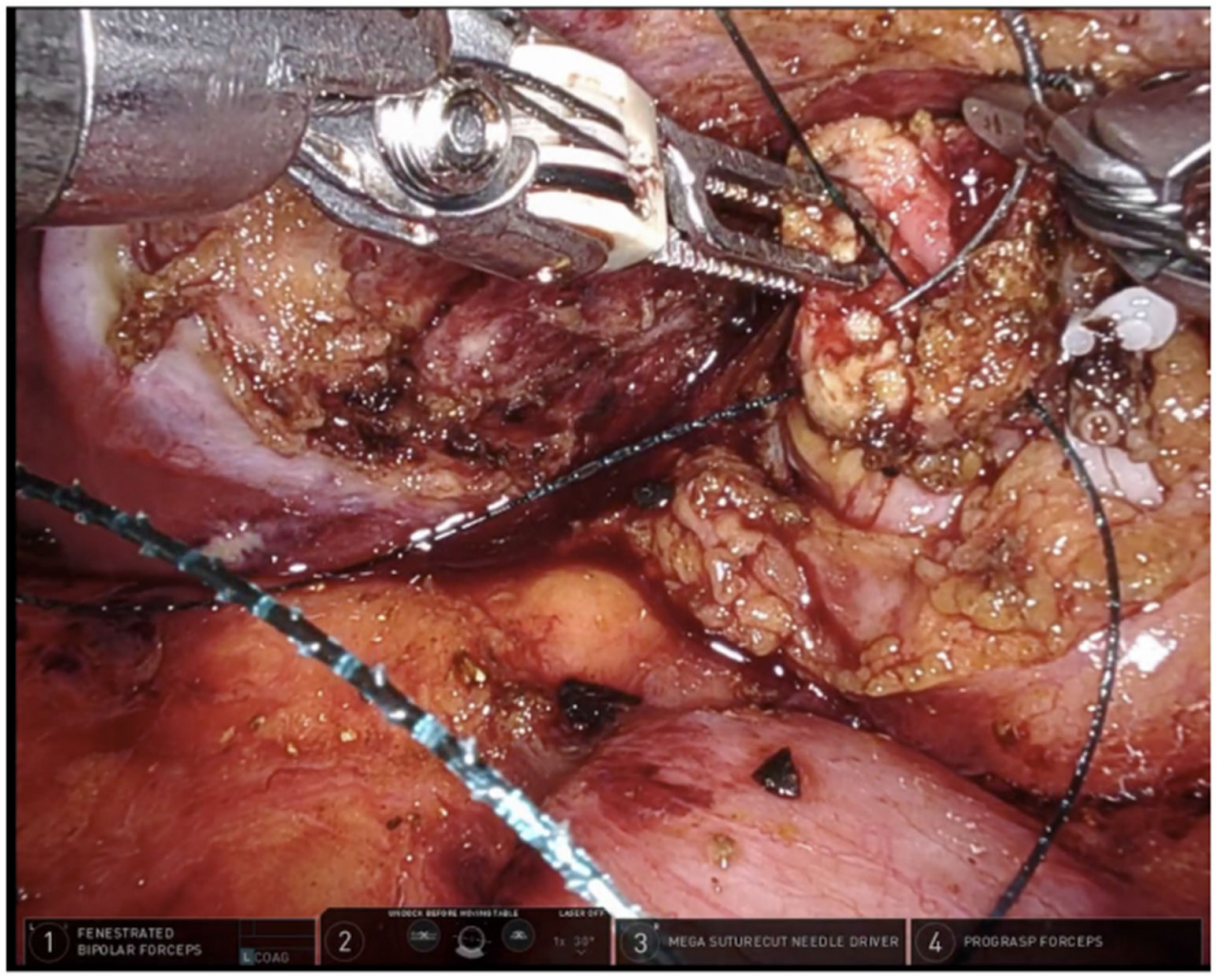
The cuff of the infundibulum was sutured from the inside, which was afforded by the dexterity and precision of the robotic instruments.

It was clear that a CVS could not be achieved, and the decision was made to proceed with a fundus-first subtotal reconstituting cholecystectomy. A laparoscopic specimen retrieval bag was placed under the infundibulum as the fundus was circumferentially dissected off the cystic plate. The 4th robotic arm was brought in from under the left rib cage at the left anterior superior iliac spine and retracted the liver. The liver was protected by a portion of a radiographically tagged sponge to diffuse the pressure of the robotic grasper and avoid liver injury. After the gallbladder was placed in the specimen bag, the infundibulum was opened, and ten 1-cm stones were removed without spillage. The wristed instruments helped to avoid crushing gallstones or spilling stones or bile into the peritoneal cavity. Clear bile emanated from the orifice, suggesting an increased likelihood of biliary fistula if the cystic duct was left open [Figure 3]. The infundibular cuff was sutured from the inside to create a 2 cm pouch with a 3-0 V-lock suture in two layers using a deep horizontal mattress and then superficial running stitch, which was easily accomplished due to the dexterity and articulation of the robotic arms [Figure 4]. A radiographically tagged sponge was left over the infundibular pouch while hemostasis was obtained and ICG reassuringly confirmed there was no bile leaking from the pouch. A drain was left in place in the hepatorenal recess out of an abundance of caution. The patient recovered from anesthesia and was discharged from the Post-Anesthesia Care Unit (PACU) in stable condition. She returned to the clinic on postoperative day 5 for drain removal. Pathology showed chronic cholecystitis.

### Case presentation 2: RAL fundus-first subtotal fenestrating cholecystectomy

**Figure 5.**
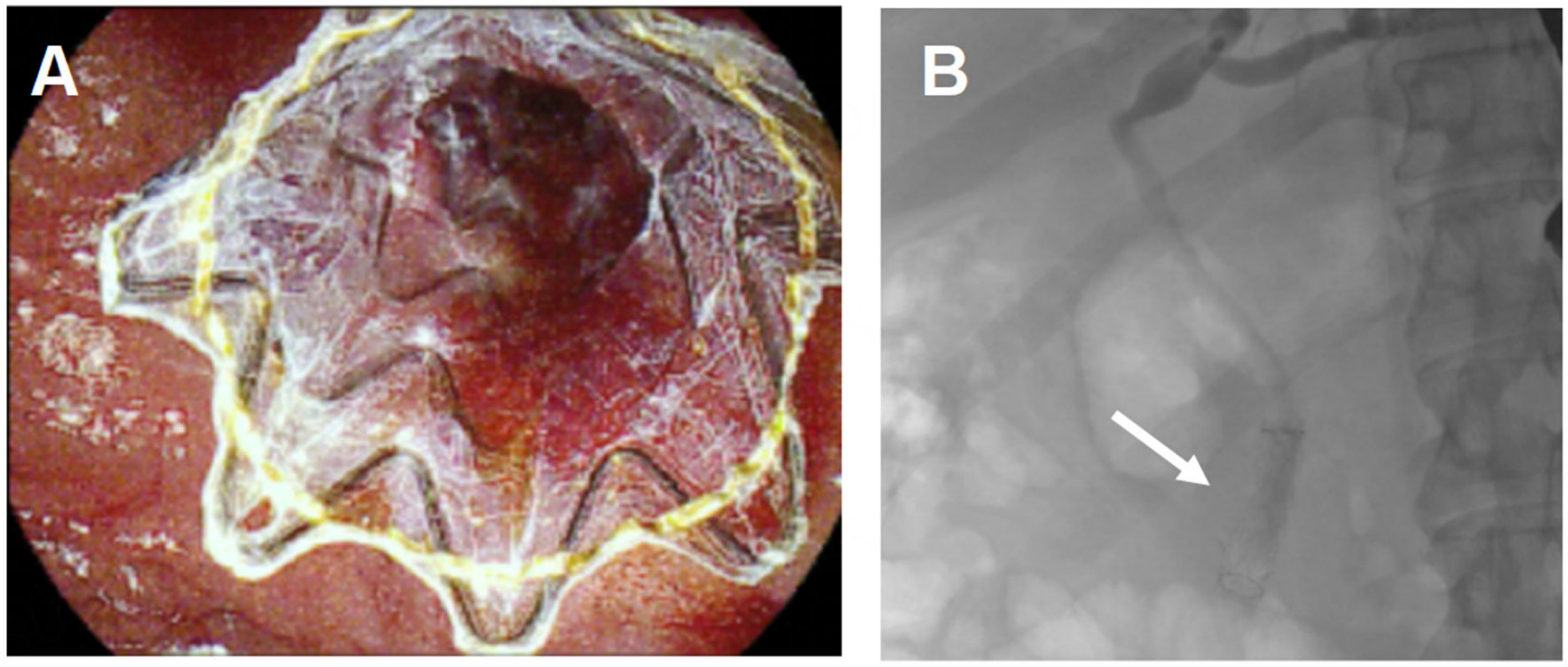
A covered metal stent was placed during an ERCP for tamponade of blood oozing at the biliary orifice. (A) The stent was observed using the duodenoscope (B) and fluoroscopically during the procedure.

**Figure 6.**
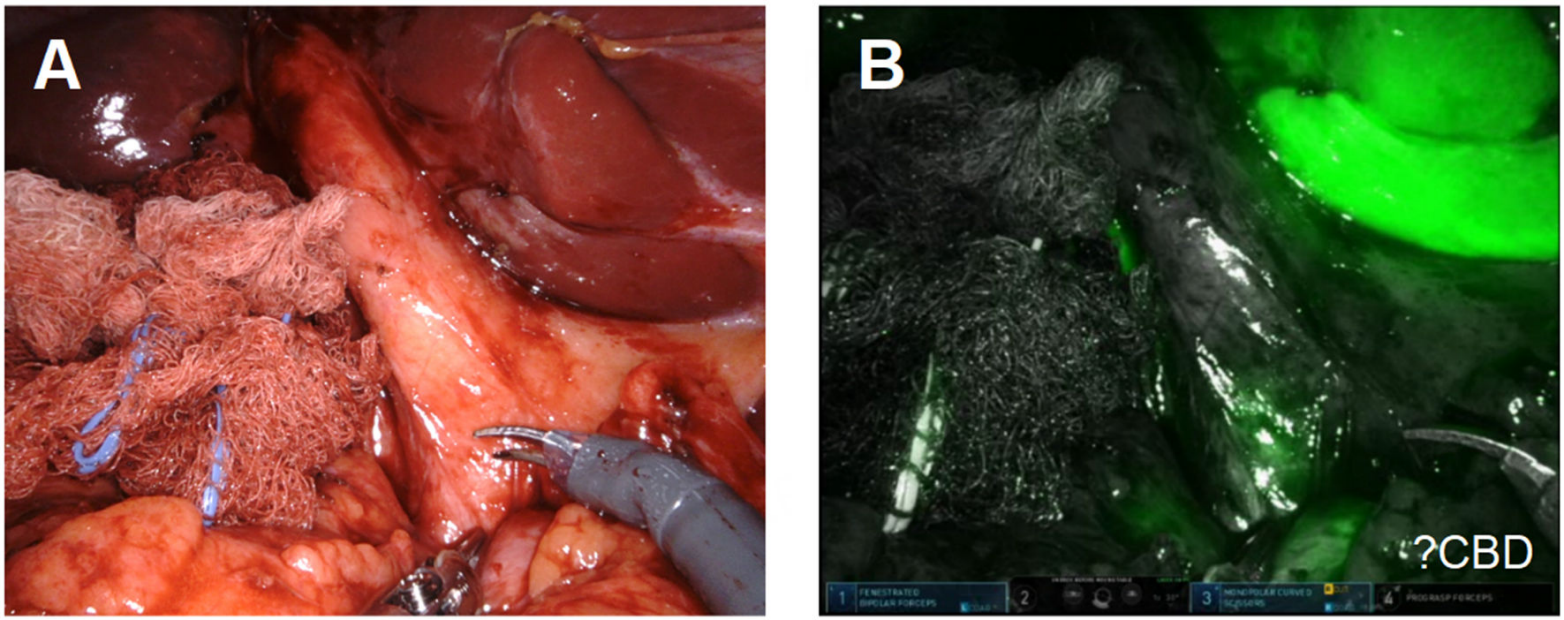
ICG was used to assess the biliary anatomy. The infundibulum appeared fused to the presumed CBD seen using (A) white light illumination and (B) ICG cholangiography.

A 52-year-old male patient with hypertension and morbid obesity presented with choledocholithiasis requiring multiple ERCPs. During the last procedure, the 1.1 cm CBD stone was successfully removed with lithotripsy and balloon sphincteroplasty, and a covered metal stent was placed for tamponade of blood oozing [Figure 5]. This procedure was complicated by E. coli bacteremia on postoperative day 1. After he recovered, he was taken for RAL cholecystectomy. There were extensive adhesions between the omentum and transverse mesocolon and the gallbladder. We worked laterally to get down the Gerota’s fascia and then medially to accurately identify the gallbladder. We cleared the omentum off the porta hepatis and noted an extensive fibrofatty scar anterior to the CBD obscuring most of the view. The infundibulum appeared to be fused to a tubular structure we presumed to be the CBD [Figure 6].

**Figure 7.**
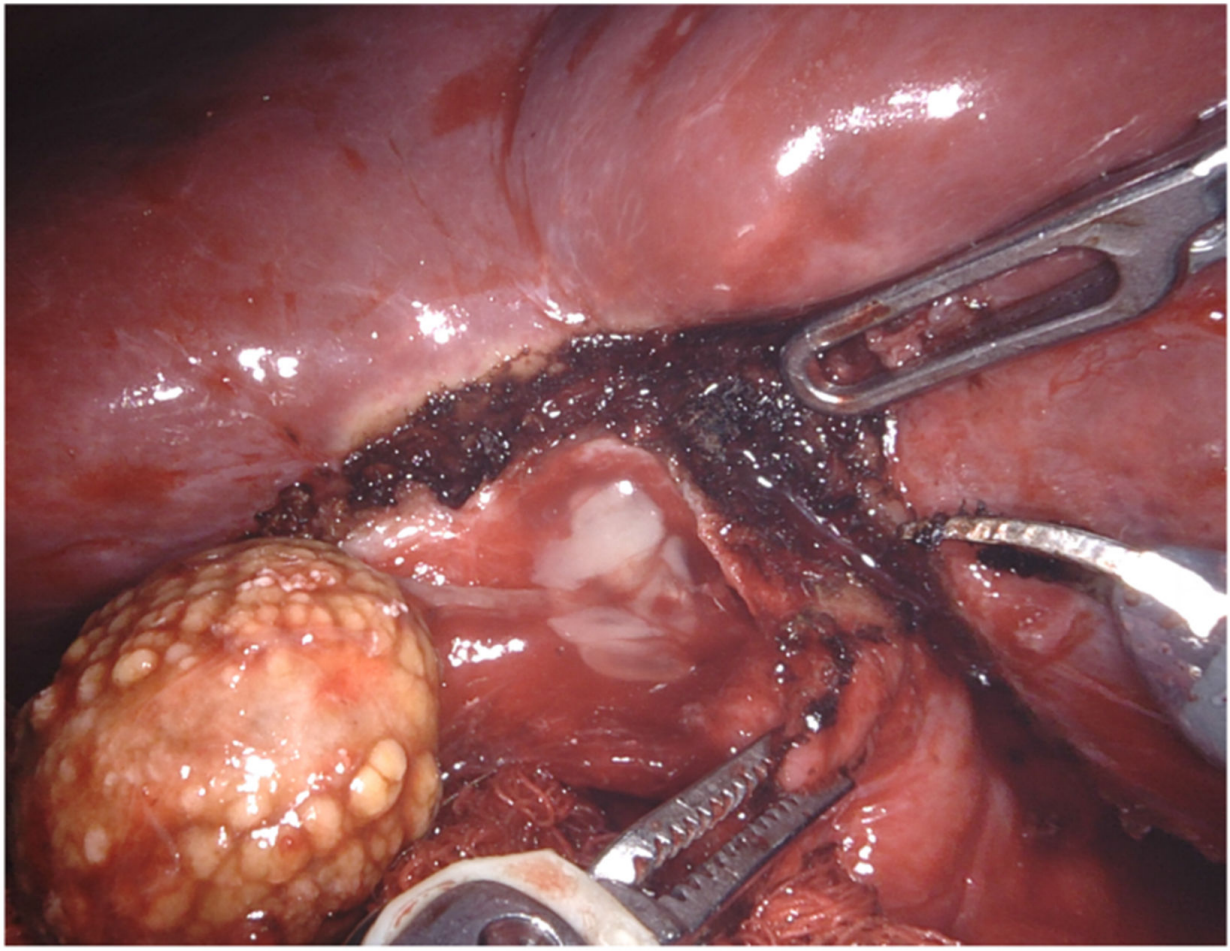
“White” bile was observed in the cystic duct orifice reflecting chronic obstruction of the cystic duct, in this case by a biliary stent and a large cholesterol gallstone.

The biliary anatomy was unclear, and no plane could be developed, so we proceeded fundus-first. The gallbladder fundus was severely inflamed. A fundectomy was performed to retrieve a large gallstone and the classic finding of “white” bile was identified, suggestive of persistent cystic duct obstruction, as expected with the prior placement of the covered metal stent [Figure 7].

**Figure 8.**
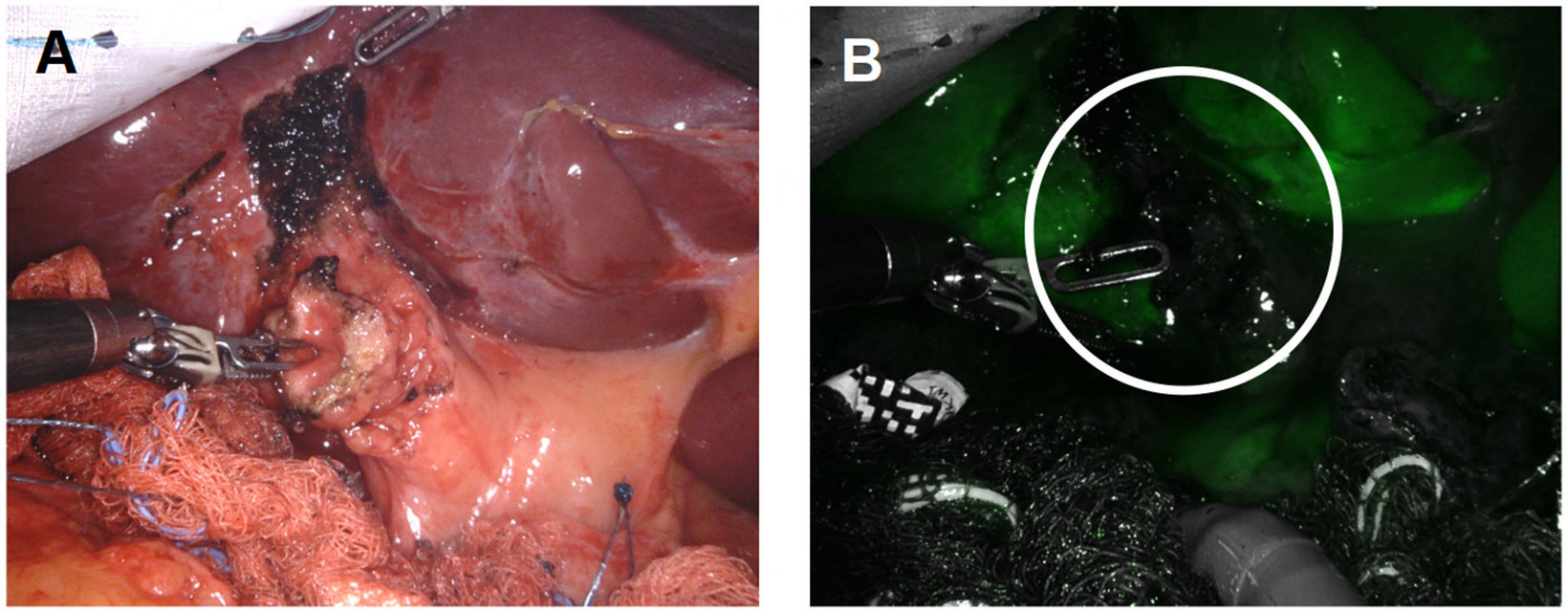
We confirmed the absence of bile leakage (A) using white light illumination (B) ICG cholangiography was used to confirm no bile leakage from the stump (white circle).

All retrieved gallstones and resected portions of the gallbladder wall were placed in a laparoscopic retrieval bag. After the removal of the large gallstone, it became clear that the infundibulum remained fused to the CBD, likely due to the covered metal stent. The tissue quality in this area was thick and fibrotic and would likely not hold a suture well, so we left the infundibulum open and placed a drain in the right hepatorenal space. In the case of a bile leak, this would create a controlled biliary fistula, although that was unlikely given the presence of the covered stent and the absence of bile from the cystic duct orifice [Figure 8]. The patient was discharged from the PACU in stable condition. Postoperatively, there was no bile in the drain and it was removed. The patient’s covered metal stent was removed 5 weeks later during an ERCP. Pathology showed chronic cholecystitis.

### Case presentation 3: cholecystocolonic fistula managed by RAL subtotal fenestrating cholecystectomy

**Figure 9.**
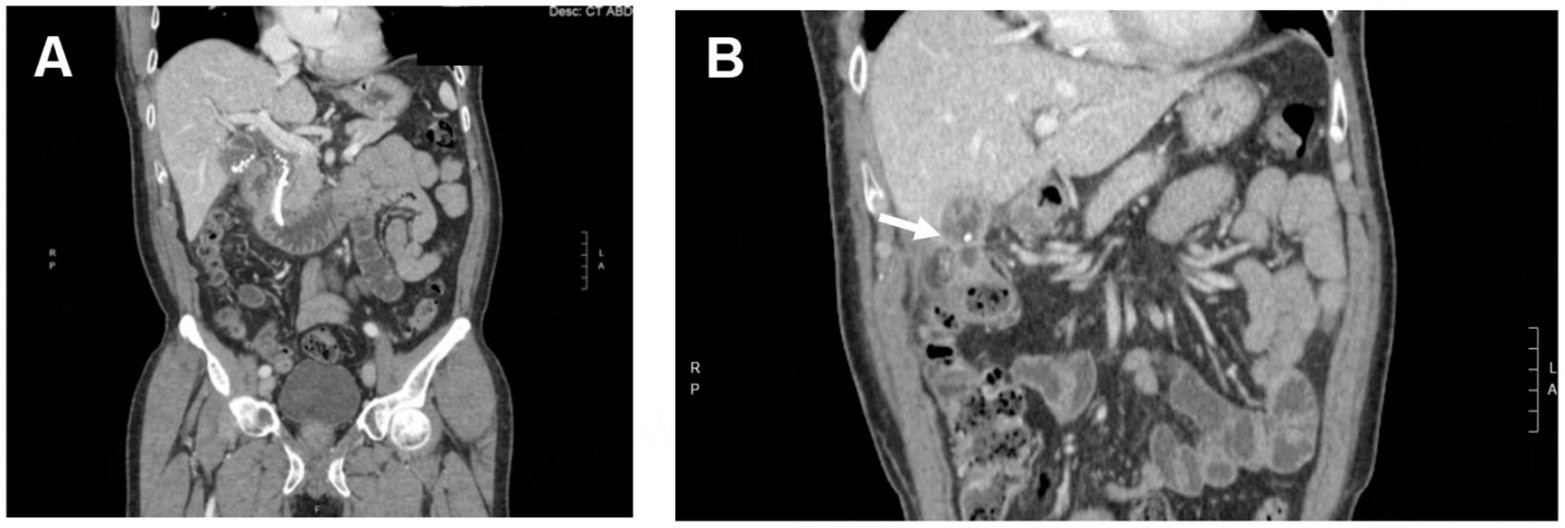
(A) Gallstones in the gallbladder and common bile duct and a stent are observed on a radiograph; (B) a cholecystocolonic fistula is observed (white arrow) on a radiograph.

**Figure 10.**
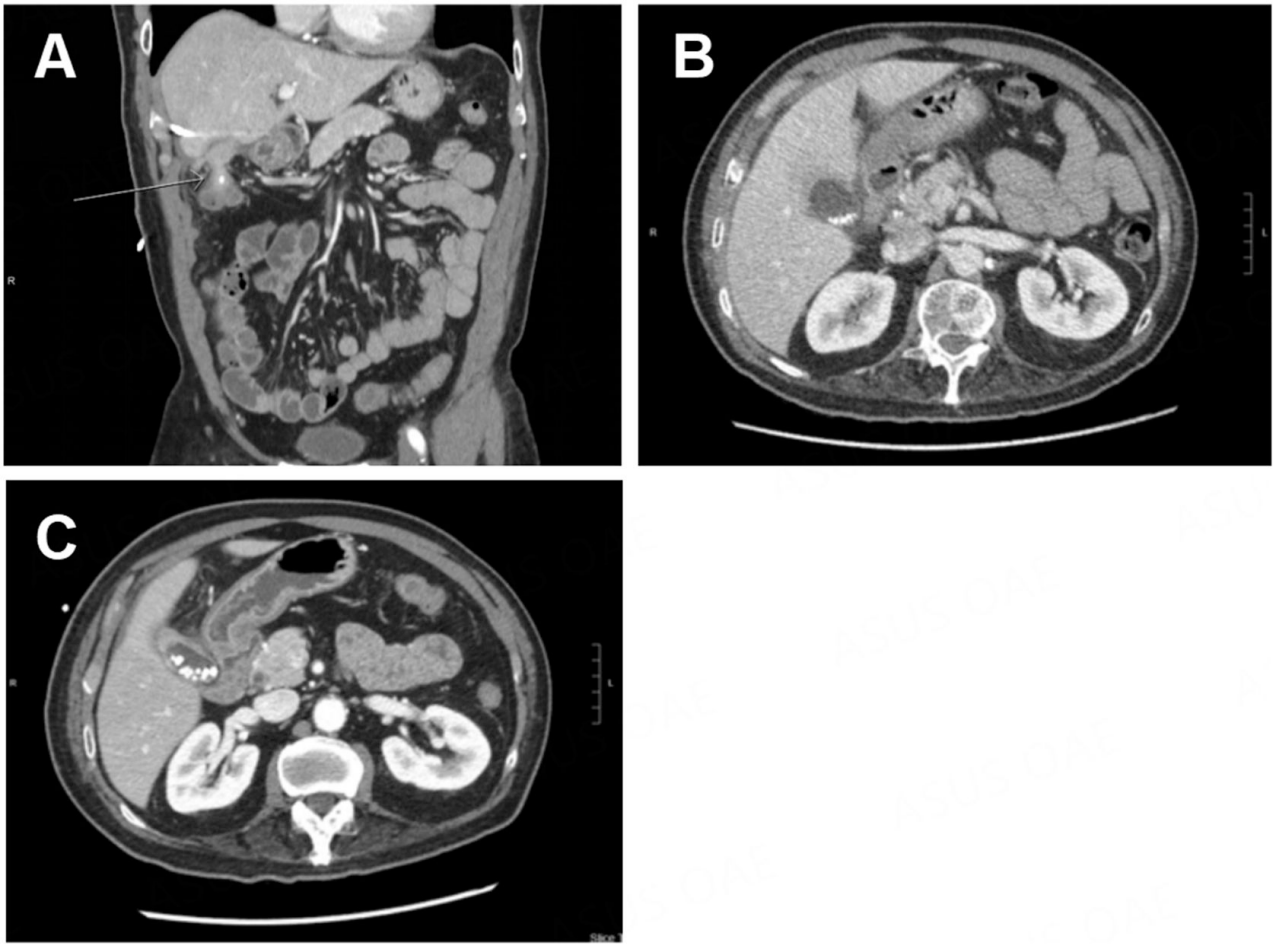
(A) A persistent cholecystocolonic fistula is observed (white arrow) after placement of a percutaneous cholecystostomy tube; (B) acute cholecystitis is observed before placement of a percutaneous cholecystostomy tube; (C) several weeks after the cholecystostomy tube was placed, improvement in GB inflammation was observed.

A 65-year-old male patient with hyperlipidemia and non-ST-elevation myocardial infarction requiring percutaneous coronary intervention presented to an outside hospital with nausea, vomiting, and abdominal pain and was found to have choledocholithiasis complicated by cholangitis. He underwent an ERCP that demonstrated suppurative cholangitis with a single stone in the CBD; because he was on dual antiplatelet therapy, the stone was not removed, and instead, a CBD stent was placed [Figure 9A]. During that admission, he underwent an attempted laparoscopic cholecystectomy which was aborted due to extensive inflammation in the right upper quadrant (RUQ). He was transferred to our institution for a higher level of care. Radiographs showed a fistula between the gallbladder and colon, with a gallstone passing through the wall [Figure 9B]. He then suffered another episode of acute cholecystitis, and the decision was made to have interventional radiology (IR) place a percutaneous cholecystostomy tube. Imaging again confirmed the persistence of his cholecystocolonic fistula [Figure 10A]. After a few weeks, there was radiographic evidence of improvement of inflammation around the gallbladder and possible resolution of the fistula [Figure 10B and C]. He was taken for RAL cholecystectomy.

**Figure 11.**
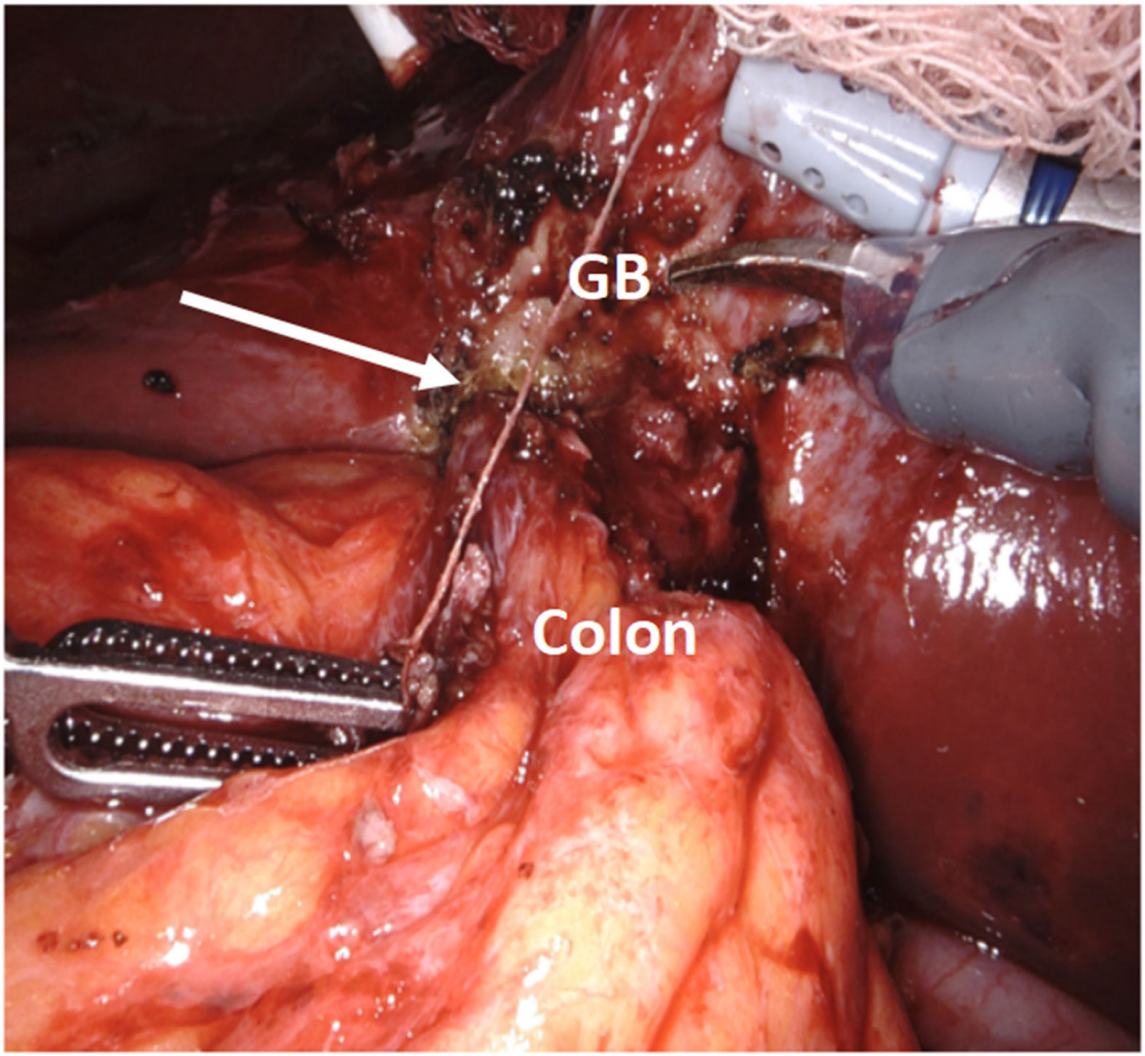
The gallbladder fundus was fused to the colon.

As is typically the case, the natural positioning of the cholecystostomy tube prevented adequate retraction of the gallbladder fundus towards the right shoulder; thus, the cholecystostomy tube was divided to gain mobility and improve retraction. The dissection began laterally by taking down the omentum carefully. As predicted, the colon was seen to be fused to the gallbladder fundus at the site of the suspected fistula [Figure 11].

**Figure 12.**
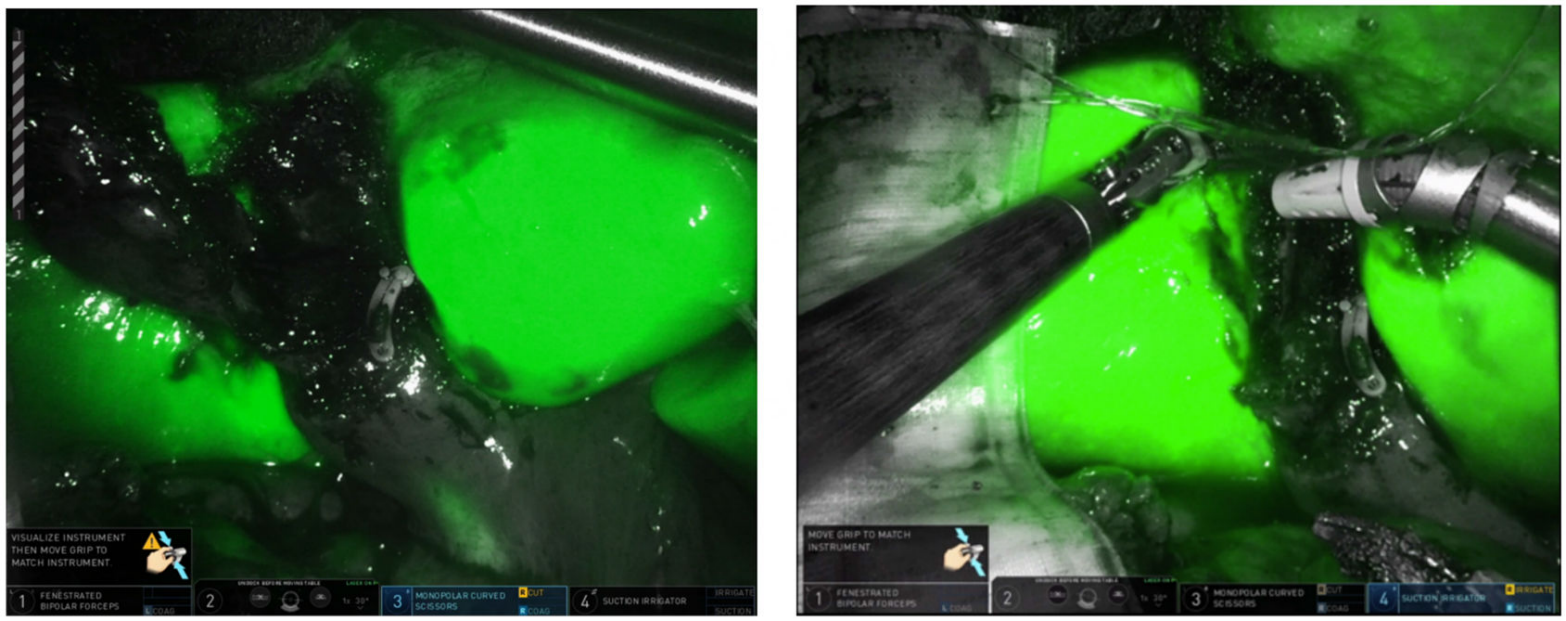
(A) ICG was used to assess biliary anatomy; (B) no ICG was identified as leaking from the cystic plate.

With minimal dissection, we determined that the duodenum was free of the infundibulum. We first incised the fundus anterior to the site of the fistula and examined that site from inside the lumen of the gallbladder. We planned to leave a divot of gallbladder wall on the colon that we could use to close the fistula with running 3-0 barbed delayed absorbable suture or, if the fistula had spontaneously closed after stone passage, just leave the divot as a patch on the colon. After the creation of the divot, we closed the body of the gallbladder with a 3-0 suture to complete the cholecystectomy. ICG cholangiography was used interactively during the dissection and we identified that the bottom third of the cystic plate was drawn into the porta hepatis in the region of the right hepatic duct, so we did a subtotal cholecystectomy, leaving the entire medial infundibulum as a protective barrier to the ductal system [Figure 12]. In this case, there was no succus or bubbling of the divot of gallbladder wall on the colon to indicate a patent fistula that would require repair. Our planned intraoperative colorectal surgery consult confirmed the findings and agreed that the gallbladder wall divot appeared to be an adequate patch to the fistula. ICG confirmed no bile leak from the cystic plate. Out of an abundance of caution, a drain was left in the hepatorenal recess.

He was discharged from the PACU, but 8 days later, he had abdominal pain, nausea, and vomiting and was found to have a deep space surgical site infection in the gallbladder fossa not in communication with the drain. The drain was repositioned by IR. When the drain output became serous and the volume decreased, the drain was removed by IR per protocol. Pathology showed acute and chronic cholecystitis.

### Case presentation 4: combined endoscopic and RAL management of iatrogenic cholecystoduodenal fistula

**Figure 13.**
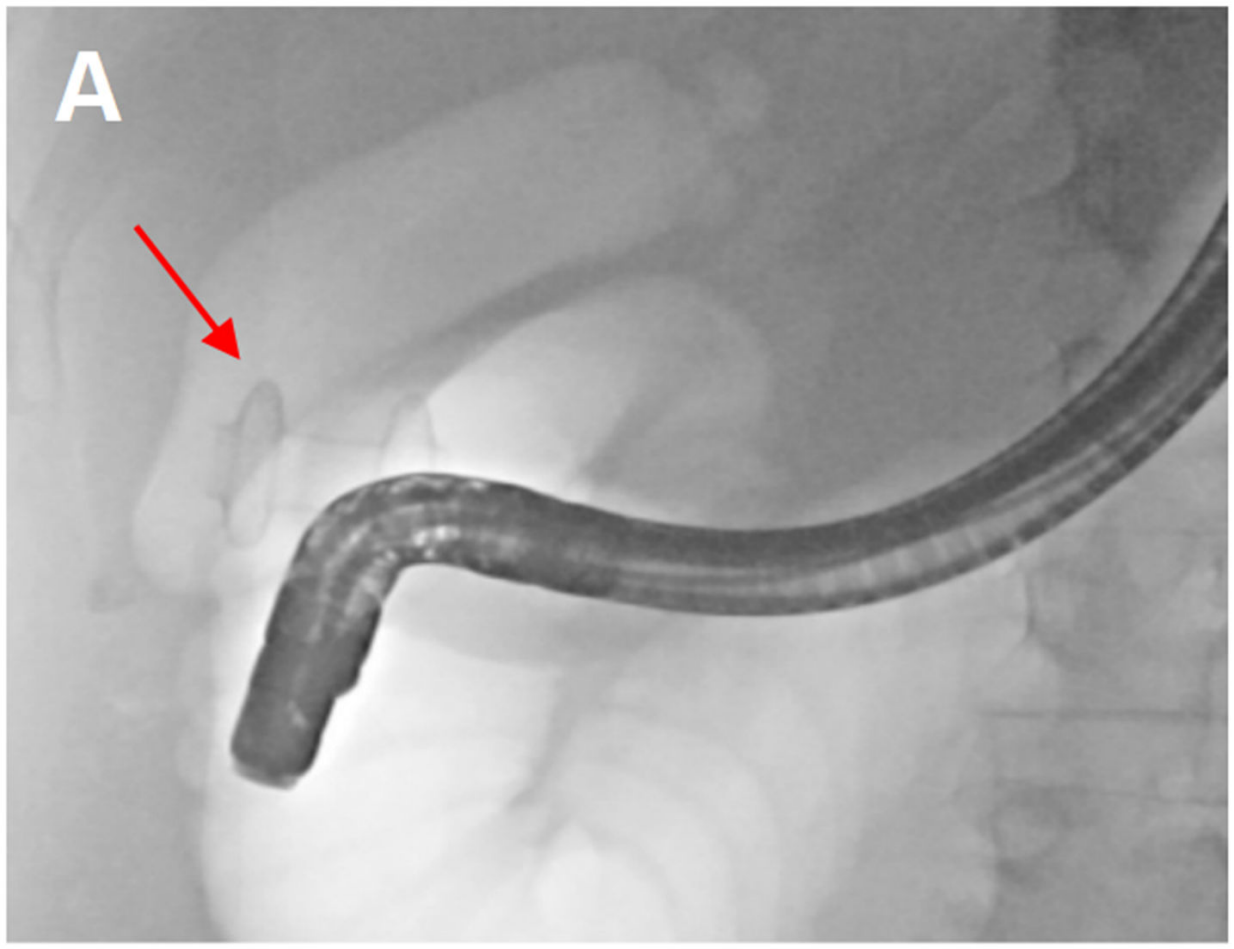
(A) lumen-apposing metal stent between the gallbladder and duodenum was seen during an ERCP (red arrow).

A 55-year-old female patient presented at a community hospital in 2016 for RUQ pain, cholelithiasis, and CBD dilation to 9 mm with a suspected mass seen on outpatient imaging. During an EUS done locally, an ampullary mass was suspected. They were unable to cannulate the CBD and biopsies were non-diagnostic. Based on concerns about periampullary malignancy and to address the CBD dilation and RUQ pain, they placed a 10 mm × 10 mm lumen-apposing metal stent from the gallbladder body to the first portion of the duodenum [Figure 13].

**Figure 14.**
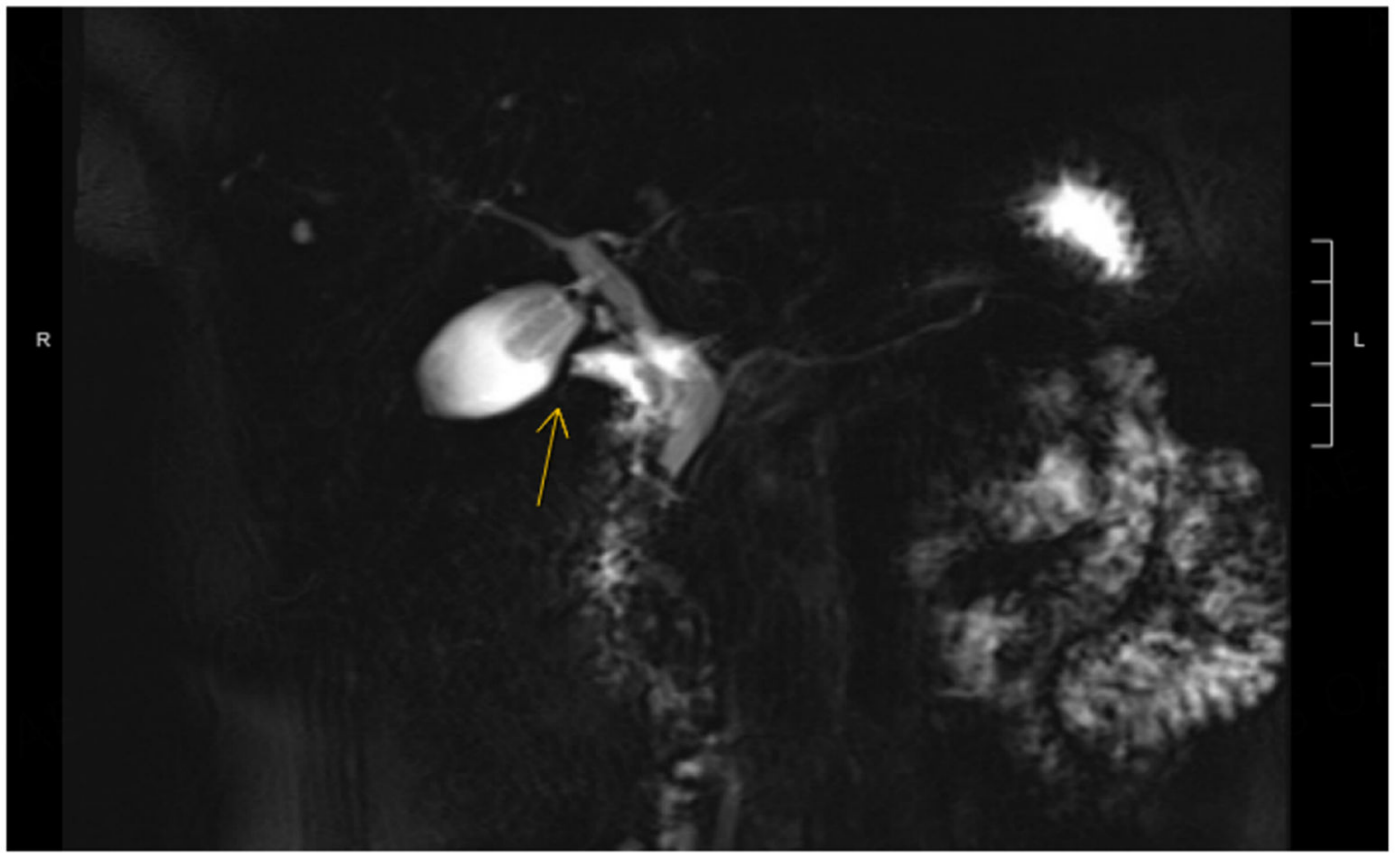
Magnetic resonance cholangiopancreatography (MRCP) with secretin and abnormal tethering of D1 to the gallbladder was observed (yellow arrow).

The RUQ pain improved with gallbladder decompression and the patient was referred to us for evaluation of the possible periampullary malignancy. Repeat EUS found no periampullary mass and confirmed a diagnosis of benign papillary stenosis. The gallstones had passed, and the gallbladder was now empty, with the cholecystoduodenal stent in place. After a multidisciplinary discussion, we opted to remove the stent to avoid erosions, bleeding and other long-term sequelae of this type of stent that is intended for short-term use. We planned to delay her cholecystectomy until she developed recurrent gallstones and symptoms, as the recent cholecystoduodenal stent would increase her chance of duodenal injury. Five years later, she developed severe RUQ pain that prompted an ER visit, with ultrasound showing cholelithiasis and dilation of CBD to 9 mm with distal tapering. A magnetic resonance cholangiopancreatography (MRCP) showed no neoplasm and the first portion of the duodenum appeared tethered to the gallbladder [Figure 14].

**Figure 15.**
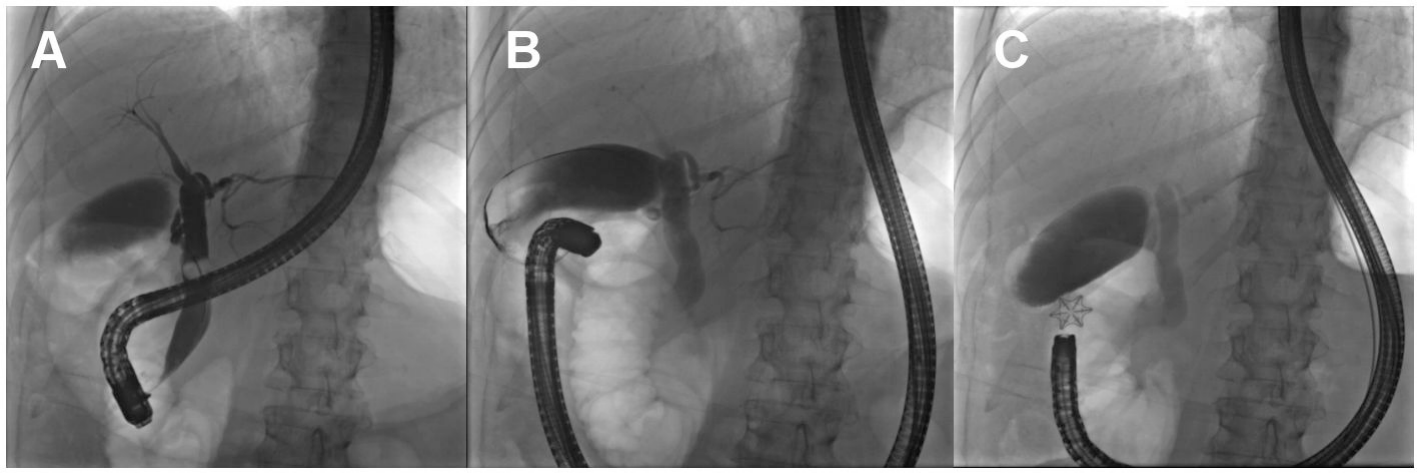
ERCP shows a patent fistula. (A) balloon occlusion cholangiogram of the CBD with contrast filling the cystic duct, gallbladder then entering the duodenum via the fistula (red circle) and (B) a wire passed retrograde through the fistula into the gallbladder demonstrates a patent fistula; (C) the ERCP patent fistula closed with a Steris Padlock clip.

There was no air in the gallbladder to suggest a patent fistula, but following a multidisciplinary discussion, we planned an ERCP to attempt endoscopic closure of the fistula if detected, prior to operative intervention. During the ERCP, the duodenal bulb was investigated and there was a clear persistent fistula with bile flowing into the first portion of the duodenum [Figure 15]. A wire was passed through the fistula to the gallbladder to confirm its presence. Using twin graspers and a Steris Padlock clip, the fistula was closed using a piece of the gallbladder wall as a buttress.

**Figure 16.**
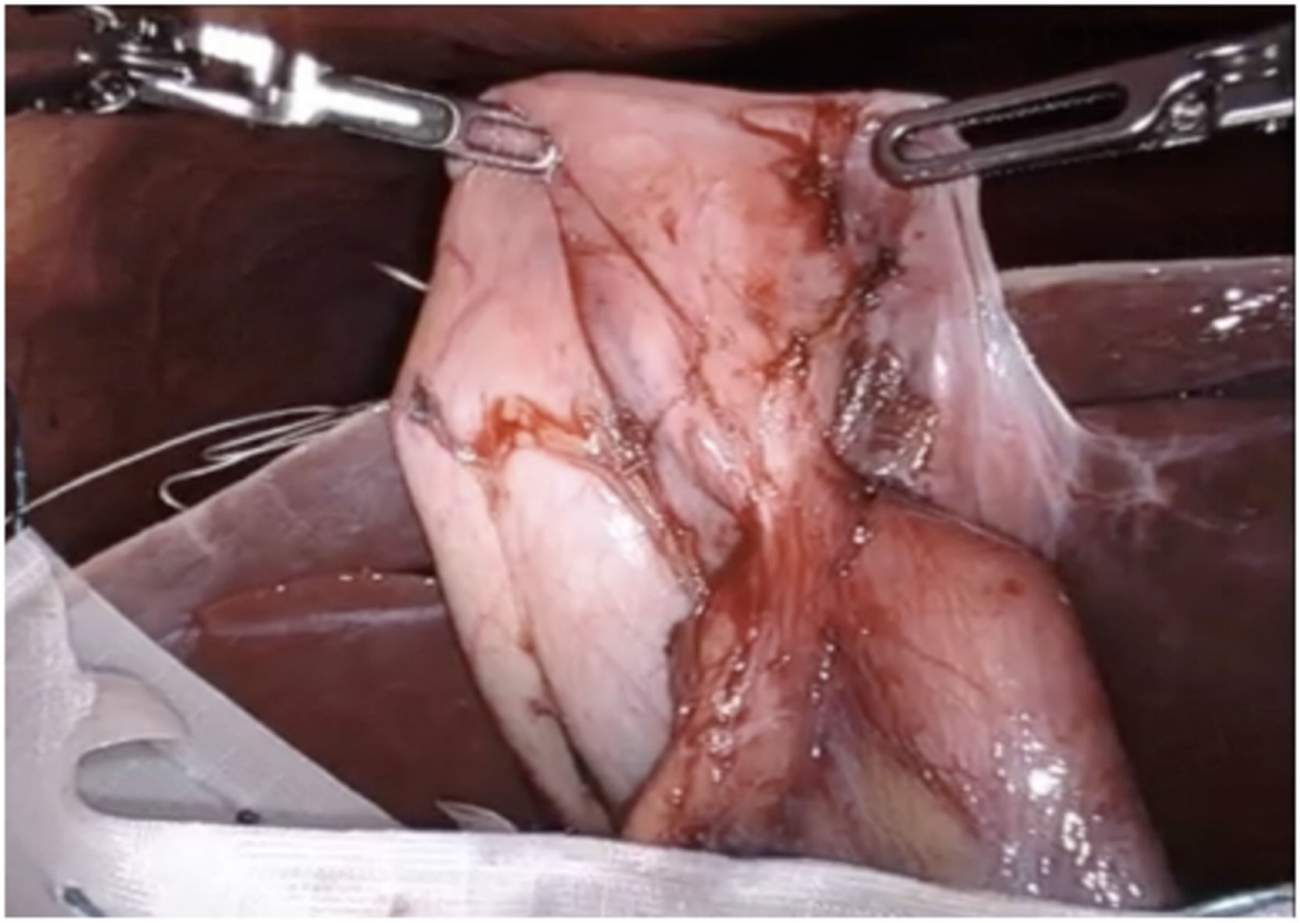
Fusion of gallbladder to duodenal wall.

**Figure 17.**
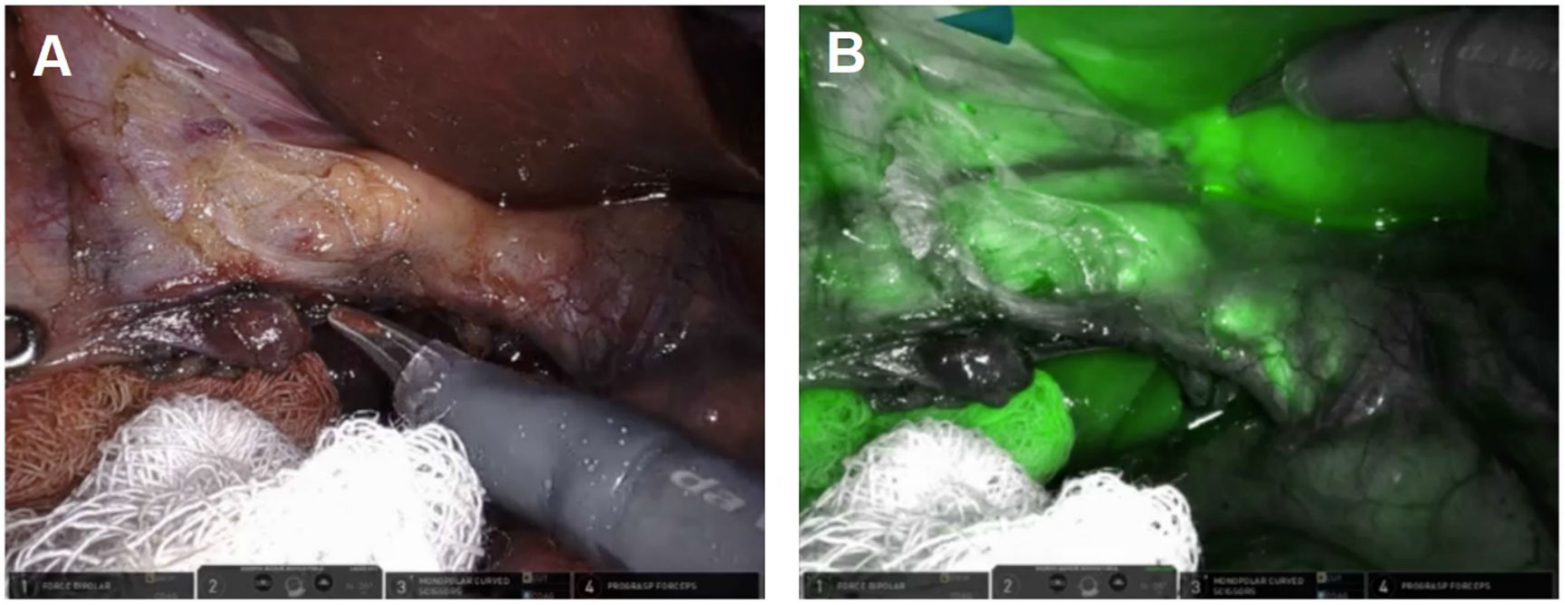
Take off of the cystic duct and pulsatile cystic artery were observed using (A) white light illumination and (B) ICG cholangiography.

**Figure 18.**
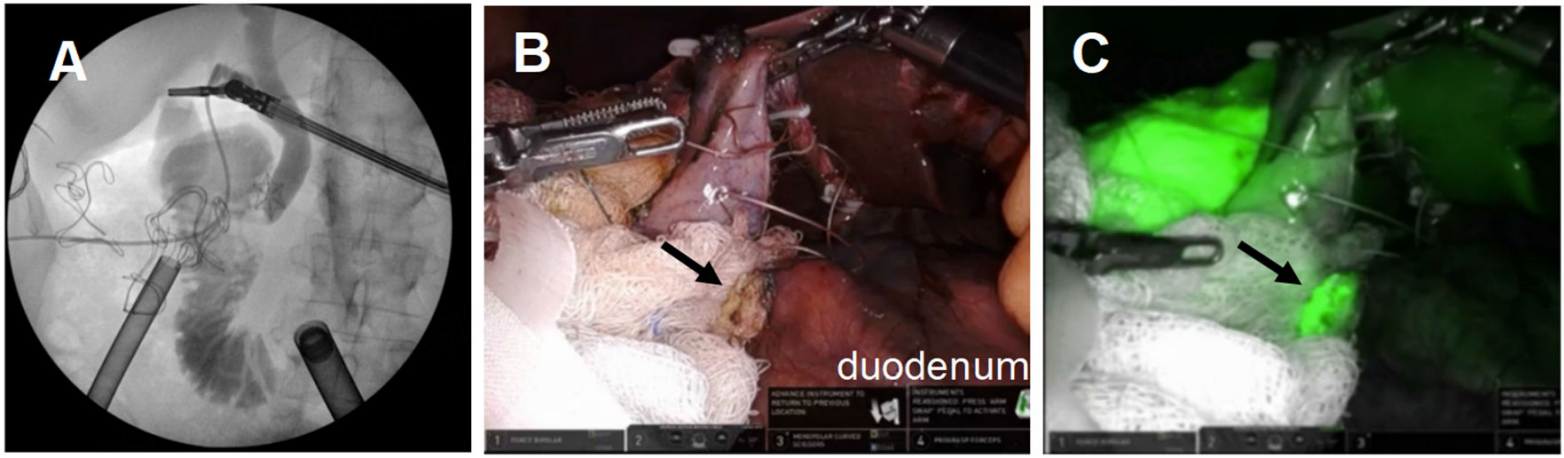
Confirmation of closure of the cholecystoduodenal fistula. (A) Intraoperative cholangiogram showed no leakage of bile from the duodenum; (B and C) ICG was used to confirm the absence of bile leakage accumulation in the operative field from the GB mucosa patch (black arrows) on the duodenum.

During RAL cholecystectomy, a knuckle of the duodenum was fused to the gallbladder, as expected, with the stellate-shaped clip’s effect visible on the duodenal wall without exposed metal [Figure 16]. To avoid duodenal injury, a divot of the gallbladder was excised and left on the duodenum. The gallbladder defect was sutured close to avoid bile spillage that would confound our interpretation of ICG in the field. Using ICG cholangiography, we identified the CHD and the takeoff of the cystic duct and the pulsatile cystic artery [Figure 17]. The robot aided in the precise skeletonization of the junction of the infundibulum and cystic duct to restore normal anatomy. These findings were used to confirm the CVS, and the gallbladder was successfully resected. The absence of accumulated ICG and bile using a fluoroscopic intraoperative cholangiogram confirmed that bile was not leaking from the prior fistula [Figure 18]. A 19-French soft silicone drain was left in the hepatorenal space adjacent to the duodenal repair. The patient did well postoperatively, and the drain was removed on postoperative day 5. Pathology showed cholelithiasis without cholecystitis.

## DISCUSSION

The robot was a helpful tool in our management of these difficult gallbladders. Tao *et al.* found that robotic cholecystectomies had a shorter hospital length of stay, less estimated blood loss, and less conversion to open compared to laparoscopic cholecystectomies^[13]^. In their series, thirty-day overall morbidity and total operating room time were similar between groups. However, because surgery for benign gallbladder disease generally has low complication rates, low blood loss, and quick recovery, results like these are not consistent^[14,15]^. For minimally-invasive surgeons comfortable with using the robot, they may preferentially use it to manage the complex biliary disease, so randomized control trials between robotic and laparoscopic surgery are not practical.

The robotic approach provides value to other complex minimally-invasive biliary surgeries. Magge *et al.* describe the definitive management of 6 cases of Mirizzi syndrome with the robot. They perform careful dissections and Roux-en-Y hepaticojejunostomy reconstruction for three patients without conversion to open^[16]^. Similarly, Marino *et al.* successfully performed robotic-assisted hepaticojejunostomy for iatrogenic bile duct injuries after laparoscopic cholecystectomy. They managed eight E2 injuries, two E1, and one E3 and E4 bile duct injury^[17]^. Both authors found that the robotic platform improved their delivery of care with selective delivery of energy, enhanced visualization, sophisticated instrumentations, filtering out hand tremors leading to improved hand-eye coordination, and surgeon endurance and comfort.

We had similar experiences with these four cases that highlight the principles of operative management of difficult gallbladders. The surgeon’s ability to perform a cholecystectomy safely without conversion to an open operation is augmented by the use of the robotic platform, including the use of ICG and integrated fluorescent imaging to evaluate biliary anatomy, superior optics and improved dexterity and precision.

The algorithm of operative decision making is illustrated in these four cases: predicting the success of safe dissection of the hepatocystic triangle to obtain the CVS, strategic use of a fundus-first dissection to avoid injury to nearby structures, and pivoting to subtotal fenestrating or reconstituting cholecystectomy when the CVS cannot be obtained. Dynamic use of ICG cholangiography facilitates a safe dissection of the hepatocystic triangle and infundibulum. In our experience, if the infundibulum and CHD move together with lateral retraction, the structures are likely fused, and it would be unwise to pursue an aggressive dissection in the region of the hepatocystic triangle. Instead, transitioning to a fundus-first subtotal cholecystectomy and transection of the gallbladder above the hepatocystic triangle affords the opportunity to leave the infundibular cuff adherent to the CHD and avoid bile duct injury. Once the gallbladder is open, it is important to remove all the stones and carefully place them in a specimen bag. Our preference is then to determine if bile emanates from the cystic duct orifice, which is easily seen inside the empty infundibulum or in pools of accumulated ICG. In such cases, our preference is to close the infundibular cuff, usually leaving a < 2.5 cm cuff, which is straightforward with the dexterity of the robotic instruments using barbed absorbable sutures. We have not seen leaks or recurrent gallstones with this approach in 20 cases over 4 years (unpublished observations). If there is no bile emanating from the cystic duct orifice, simple drainage is adequate. We have not seen postoperative biliary fistulas in these patients. In the rare case in which a subtotal fenestrating resection is done and bile is seen intraoperatively via the cystic duct orifice but neither the cuff nor the orifice can be closed due to poor tissue quality, we leave a 12Fr red Robinson catheter inserted into the cystic duct orifice and a 19-French drain in the hepatorenal fossa. The patient remains in the hospital overnight, and if there is bile in either drain the next day, an ERCP is performed and two plastic stents or a covered metal stent are placed. The red Robinson catheter is then capped prior to discharge and both drains are removed once the fluid is non-bilious, typically within 2 weeks.

In these cases, surgical and gastroenterology colleagues were a readily used resource to solve unusual gallbladder problems including the cholecystoduodenal fistula and the cholecystocolonic fistula. The importance of the multidisciplinary team cannot be understated.
